# Case report: Patients with positive HER-2 amplification locally advanced gastroesophageal junction cancer achieved pathologic complete response with the addition of pembrolizumab to chemotherapy plus trastuzumab as neoadjuvant therapy

**DOI:** 10.3389/fimmu.2025.1555074

**Published:** 2025-02-12

**Authors:** Huanhuan Li, Chao Ren, Donghai Cui, Tao Wu, Zhiyong Nie

**Affiliations:** Department of Oncology, Anyang Tumor Hospital, The Affiliated Anyang Tumor Hospital of Henan University of Science and Technology, Anyang, China

**Keywords:** HER-2, immunotherapy, neoadjuvant therapy, gastric cancer, gastroesophageal junction adenocarcinoma

## Abstract

**Background:**

Human epidermal growth factor receptor 2(HER-2) is the most prominent therapeutic target for gastric (G)/gastroesophageal junction (GEJ) cancer. Guidelines recommend its use for treating G/GEJ cancers. However, targeted therapy did not significantly improve survival outcomes compared to those with neoadjuvant therapy. The KEYNOTE-811 trial revealed an improved objective response rate (74% vs. 52%; P=.0001) and median duration of response (10.6 vs 9.5 months) with the addition of pembrolizumab (PD-1) to chemotherapy plus trastuzumab compared to that with the addition of placebo in patients with HER-2 overexpression-positive advanced adenocarcinoma. Therefore, addition of PD-1 to chemotherapy plus trastuzumab may lead to a better response in patients with G/GEJ cancer.

**Case presentation:**

A 66-year-old man was diagnosed with stage III GEJ adenocarcinoma with celiac lymph node metastasis. Immunohistochemical results indicated HER-2(3+) and PD-L1 CPS=5. The patient received three cycles of pembrolizumab plus trastuzumab and chemotherapy preoperatively and underwent radical surgery on November 22, 2022.

**Conclusion:**

Patients with HER-2-positive locally advanced GEJ cancers received PD-1 immunotherapy combined with trastuzumab and neoadjuvant chemotherapy and achieved a complete pathological response. Hence, it is a novel, highly specific, and potent therapeutic option for HER-2-positive patients and should henceforth be considered as a new treatment approach.

## 1 Introduction

Gastric cancer (GC), anatomically separated into true gastric adenocarcinomas and gastroesophageal junction (GEJ) adenocarcinomas, is one of the most common malignant tumors and one of the leading causes of cancer-related mortality in both China and worldwide (1, 2). Surgery combined with either adjuvant or neoadjuvant chemotherapy is the recommended approach for the treatment of locally advanced disease because these therapies have been shown to improve the disease-free survival and overall survival (OS) (3–5). However, among patients with locally advanced GC, the effect of the recommended treatment is unsatisfactory, with a reported 5-year OS rate of 36–78% (6–8). Therefore, additional treatments such as radiotherapy, targeted treatment, or novel immune checkpoint inhibitors have been proposed as supplements to the current therapeutic regimen in an attempt to gain further benefits.

Human epidermal growth factor receptor 2 (HER2) is an important biomarker and critical driver of GC tumorigenesis (9). HER-2 positive gastric cancer is a unique type of adenocarcinoma with positive criteria being immunohistochemistry (IHC) 3+ or fluorescence *in situ* hybridization-positive staining of tumor cells. The detection rate of HER2 positive in GC is between 17% and 20%, which is related to the histopathological type, lesion location and patient sex (10). HER2-positivity is most likely in enteric, medium-differentiated, gastroesophageal junction adenocarcinoma (EGJ) than in diffuse, poorly differentiated, non-EGJ carcinomas (11, 12). Since 2010, combination therapy with the anti-HER2 antibody trastuzumab and chemotherapy has become the standard first-line treatment for patients with HER-2-positive G/GEJ cancer (13). However, the progress in this field has slowed over the past decade. With the advent of IO, the KEYNOTE-811 study confirmed that the combination of anti-HER-2 therapy with chemotherapy and immunotherapy could further improve the objective response rate (ORR), progression-free survival (PFS), and overall survival (OS) (14). Based on the results of the KEYNOTE-811 trial, the FDA and Drug Administration approved pembrolizumab plus trastuzumab and chemotherapy as first-line treatments for HER2+ metastatic G/GEJ cancer. Targeted therapy and immunotherapy may exert synergistic effects. Currently, the pathological complete response rate of neoadjuvant chemotherapy for gastric cancer is 10-15%. Targeted therapy did not significantly improve the survival compared to that with neoadjuvant therapy. Therefore, whether a combination of immunotherapy plus trastuzumab and chemotherapy can achieve better efficacy remains to be determined, and relevant studies are ongoing. We report a case of immune checkpoint inhibition combined with trastuzumab and chemotherapy as neoadjuvant therapy for a patient with positive HER-2 amplification as neoadjuvant therapy.

## 2 Case report

**Figure 1:**
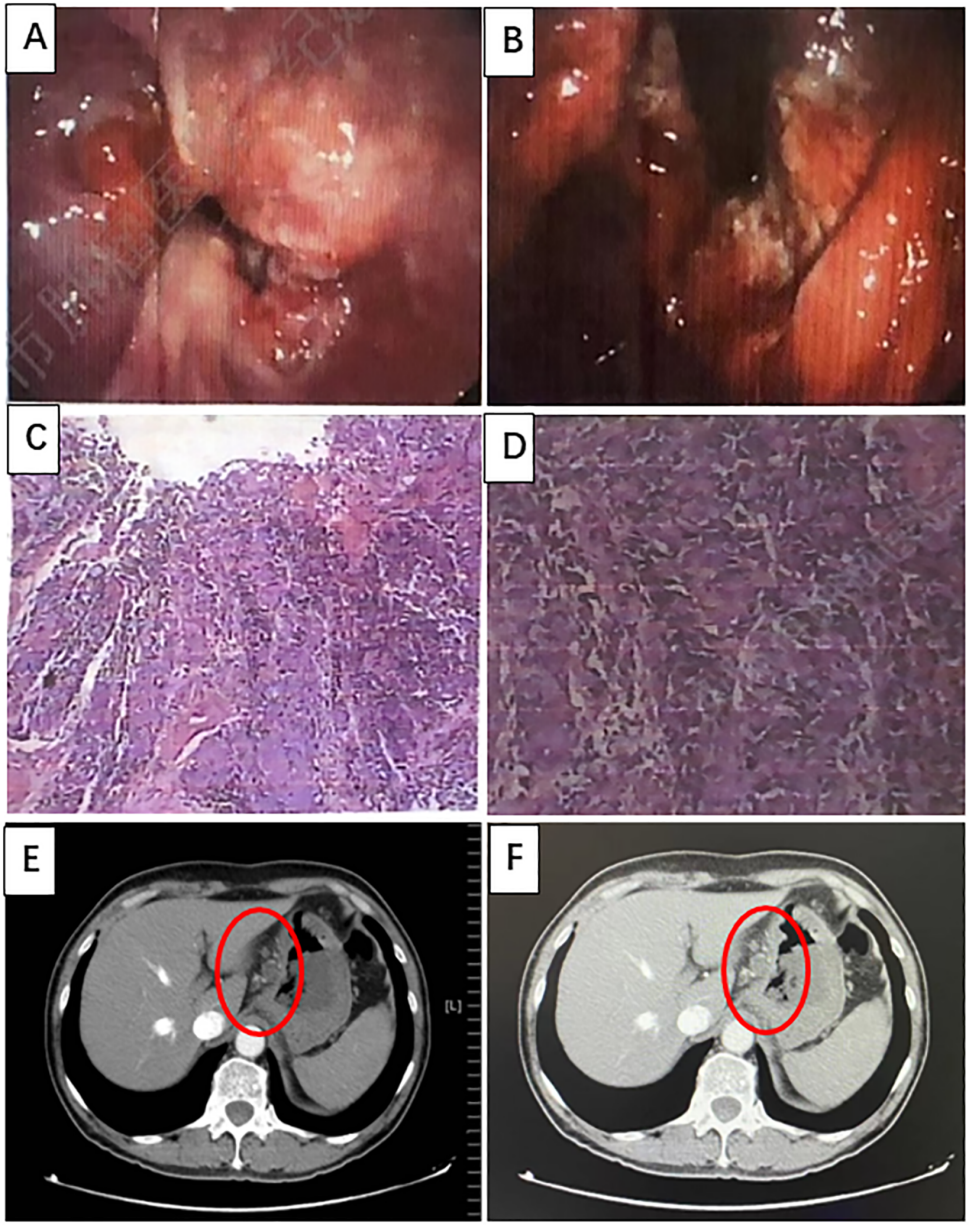
Imaging was performed at baseline, before treatment initiation. **(A, B)** Gastroscopy revealing new organisms of sunken ulcers can be seen from the whole cardia to the lesser curvature of the stomach (40–50 cm from the incisors), which can easily bleed. **(C, D)** Hematoxylin and eosin (H&E) staining of poorly differentiated adenocarcinomas (H&E, ×100 original magnification). **(E, F)** Computed tomography (CT) images of primary GEJ cancer and multiple lymph node metastases.

A 66-year-old man with no clear incentive presented with an eating obstruction in August 2022. Symptoms worsened when hard and dry foods are consumed, with no chest tightness or pain, nausea and vomiting, hematemesis and melena, fever or chills, or other discomfort. There was no history of autoimmune disease, pneumonia, interstitial lung disease, chronic obstructive pulmonary disease (COPD), hepatitis B virus (HBV) or hepatitis C virus (HCV), human immunodeficiency virus (HIV) carrier, or recent vaccination. The patient visited a local hospital on August 6, 2022 wherein gastroscopy revealed new organisms in sunken ulcers from the whole cardia to the lesser curvature of the stomach (40–50 cm from the incisor), which were easily bleeding (**Figures 1A, B**). Biopsy revealed a poorly differentiated adenocarcinoma (**Figures 1C, D**). The patient visited our hospital for further diagnosis and treatment on August 8, 2022. Contrast-enhanced Computed tomography (CT) of the abdomen revealed cardiac cancer involving the entire cardia and lesser curvature of the stomach, with abdominal lymph node metastasis (**Figures 1E, F**) with immunohistochemistry (IHC): HER-2(3+), pMMR, and PD-L1(CPS=5).

**Figure 2:**
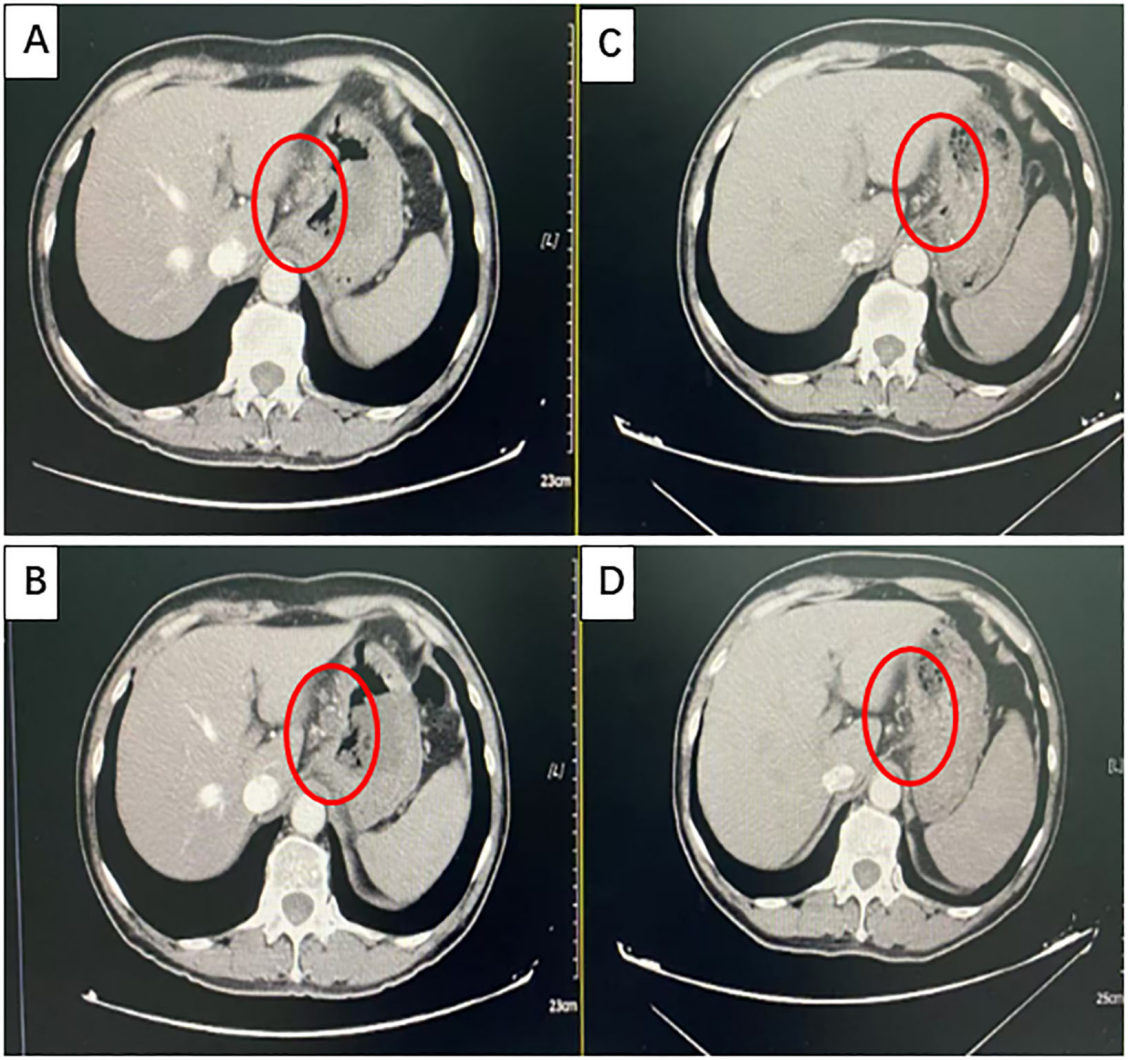
Partial response (PR) was confirmed using computed tomography (CT). **(A, B)** Images taken at baseline before treatment initiation. **(C, D)** Partial response (PR) was assessed using CT after three cycles of treatment (primary foci and abdominal lymph node metastatic lesions were markedly decreased).

**Figure 3:**
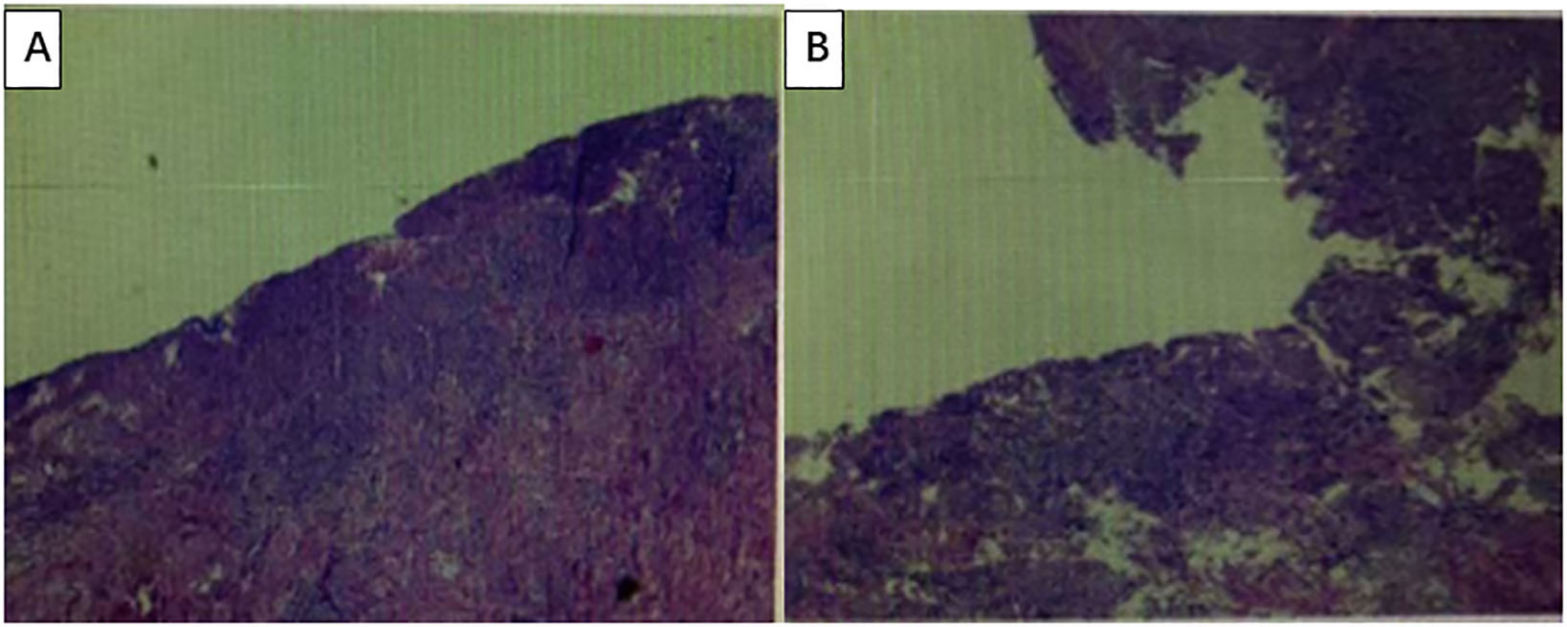
The pathological results indicated that the patient achieved a complete response. **(A, B)** Under a microscope, the surface mucosa is absent, with fibrous tissue hyperplasia, granulation tissue formation, and a large number of inflammatory cell infiltrations. No residual tumor cells were observed. No metastatic cancer was detected in the lymph nodes.

After communicating with the patient and providing comprehensive consideration, consent for neoadjuvant therapy was achieved. Three cycles of pembrolizumab +chemotherapy + trastuzumab/q21d (oxaliplatin 130 mg/m2 d1 + S-1 40 mg/m2 d1–14 + pembrolizumab 200 mg d2/q21d + trastuzumab 8 mg/kg loading dose, followed by 6 mg/kg every 3 weeks) were initiated on August 16, 2022. Partial response (PR) was assessed by CT after three cycles of treatment (primary foci and abdominal lymph node metastatic lesions were markedly decreased) (**Figures 2A–D**). Radical surgery was performed on November 22, 2022. The pathological results indicated that the patient achieved a complete response (**Figures 3A, B**). The original regimen was continued for 3 cycles after the surgery. At present, the patient is still under follow-up, and no recurrence or metastasis has been observed.

## 3 Discussion

Currently, the concept of neoadjuvant therapy has gradually become more widely accepted and appreciated because it can achieve tumor shrinkage, downstaging, improved R0 resection rates, and improved long-term survival. In China, as many as 70.8% of patients with GC are diagnosed at a locally advanced stage, with a 5-year survival rate of less than 30%. For locally advanced GC/GEJ cancers, the major guidelines still recommend neoadjuvant chemotherapy; however, neoadjuvant chemotherapy has certain limitations. For instance, the rate of pathological complete response (pCR) is approximately 10%, the 2-year disease-free survival (DFS) rate of such patients is 66.7% - 82.4%, and the 2-year overall survival (OS) rate is 70%–86.7%. Therefore, traditional neoadjuvant chemotherapy strategies require further improvement (15).

In recent years, immunotherapy has achieved great success in the treatment of advanced GC/GEJ. Some experts have proposed that immunotherapy can be advanced to neoadjuvant therapy for GC/GEJ cancer and clinical experts have made several attempts to achieve this goal. Numerous studies have confirmed that chemotherapy combined with immunotherapy can improve pCR rates in neoadjuvant therapy; however, long-term survival, such as the OS and DFS, remains controversial. Therefore, the specific regimen of chemotherapy combined with immunotherapy as a neoadjuvant therapy needs to be further optimized.

As a special group in gastric cancer, the HER-2 overexpressing population has different biological behaviors and treatment strategies compared to those in the HER-2 negative population. HER-2 positive gastric cancer is highly invasive and metastatic, and the efficacy of neoadjuvant regimen of anti-HER-2 targeted drugs combined with chemotherapy remains controversial. The PANTHERA and KEYNOTE-811 studies confirmed that a combination of anti-HER-2 therapy with chemotherapy and immunotherapy further improved the ORR (16, 17). Therefore, multiple studies are currently underway to determine whether PD-1 combined with trastuzumab and chemotherapy regimens can be used as perioperative treatment to improve efficacy. A study published in ASCO in 2022 showed that HER2 positive resectable GC/GEC receiving chemotherapy combined with camrelizumab and trastuzumab during the perioperative period had a pCR rate of 31.3% and ORR rate of 77.3% (18). In 2024, atezolizumab combined with trastuzumab and chemotherapy for perioperative treatment of HER2+ (IHC 3+ or IHC 2+/ISH+) locally advanced resectable gastric cancer or esophagogastric junction adenocarcinoma was published at ASCO-GI, and the results of the Phase II study showed that the pCR rate reached 38.1% (8/21) (19). The patient received PD-1 immunotherapy combined with trastuzumab and chemotherapy as neoadjuvant therapy and achieved pathologic complete response. This case demonstrates the clinical benefits of this protocol, which may become a new treatment option for HER-2 positive patients and further clinical trials are needed to confirm its efficacy.

## Data Availability

The original contributions presented in the study are included in the article/supplementary material. Further inquiries can be directed to the corresponding author/s.
